# The value of case reports in rare oncological scenarios: mixed method analysis of colorectal metastases from breast cancer

**DOI:** 10.1007/s10585-023-10207-9

**Published:** 2023-04-27

**Authors:** I. D. Nagtegaal, J. A. A. Snoek, P. Bult, J. Tol, S. Siesling, Q. J. Voorham, N. Hugen

**Affiliations:** 1 Department of Pathology, Radboudumc, 812, PO Box 9101, 6500 HB Nijmegen, The Netherlands; 2 PAL, Dordrecht, The Netherlands; 3 Department of Medical Oncology, Jeroen Bosch Hospital, Den Bosch, The Netherlands; 4 Department of Research and Development, Netherlands Comprehensive Cancer Organisation (IKNL), Utrecht, The Netherlands; 5 Department of Health Technology and Services Research, Technical Medical Centre, University of Twente, Enschede, The Netherlands; 6 PALGA Foundation, Houten, The Netherlands; 7 Department of Surgery, Netherland Cancer Institute, Amsterdam, The Netherlands

**Keywords:** Breast cancer, Colorectal metastases, Systematic review, Real-life data

## Abstract

With improved survival of cancer patients, we increasingly encounter infrequent metastatic locations. While for the common metastatic locations both prognostic information as well as evidence-based guidelines are available, for rare locations we have to rely on anecdotal case reports, the value of which is currently unknown. Therefore, we performed a systemic literature review and compare the results with a large national real-life cohort focussed on breast cancer patients with colorectal metastases. We performed a systematic literature search for breast cancer patients with colorectal metastases. Autopsy studies were excluded. Data on stage, histological factors, treatment and outcome were extracted. All identified cases were analysed as individual patients. The real-life cohort was extracted from the nationwide Dutch pathology databank. Linkage with the Netherlands Cancer Registry provided clinical characteristics, treatment and outcome data. Survival analyses and univariate regression were performed to identify relevant features for future treatment decisions. We identified 308 patients from 207 studies in the literature, and 454 patients in the real-life cohort. Colorectal metastases were the first metastatic event in 42.5% and 47.0% respectively. Cohorts were comparable for age, gender, location and hormone status, but differed in tumour type, stage and treatment. The time to colorectal metastases was similar in both cohorts (median of 68 months), and was dependent on presence of other metastases, nodal status, and primary breast surgery. The median overall survival after development of colorectal metastases was 20.6 months (95%CI 18.0–23.1 months). Despite a potential publication bias and lack of complete data for patients in the case report series, we have shown that an extensive systematic review can provide data that are comparable to real-life data, which can be used for decision-making and informing patients. Colorectal metastases are a late event in breast cancer patients, that is not associated with a detrimental survival.

**Supplementary Information:**

The online version contains supplementary material available at 10.1007/s10585-023-10207-9.

## Introduction

Recent improvements in oncological care result in better overall survival, even in patients with metastatic disease [1, 2]. Longer survival with metastatic disease results in the observation of new locations of metastases, that were rarely observed before, such as brain metastases in colorectal cancer patients [3]. While the conventional and most frequent locations of metastatic disease, such as liver metastases in colorectal cancer and bone metastases in breast cancer, are currently treated following evidence based guidelines [4–6], there is hardly any evidence for the efficacy of treatment for metastases diagnosed at rare sites. Moreover, while imaging is often sufficient proof for common metastatic sites, at rare sites pathology confirmation is frequently necessary.

Metastases diagnosed in the colon are relatively rare, with approximately 1 case in every 140 resections for colorectal malignancy [7]. However, even this number seems a gross overestimation, given the low numbers detected in the bowel cancer population screening programs [8]. In contrast, the small bowel is more frequently affected by metastases [9]. The mechanisms underlying these differences are not entirely clear. When focusing on metastases in the colon, various studies point towards the breast as their most common origin, supposedly responsible for 20–50% of metastases in the colon [10, 11]. Breast cancer is one of the most common types of cancer in women worldwide [12]. The prognosis of breast cancer patients, although highly dependent on subtype, is relatively good, with a 10 year crude recurrence-free survival of 81.9% [13]. Patients with metastatic disease have a 22% 5 year overall survival [14]. Numerous studies have investigated metastatic patterns of breast cancer: the most common metastatic locations are lung and pleura, bone, and liver [15]. Metastases to the gastrointestinal tract are well recognized, and occur in 0.2–11% of cases [15–18]. The incidence of colorectal metastases in autopsy studies is as high as 10% [19].

For breast cancer patients with colorectal metastases, as well as for patients presenting with other rare oncological scenarios, there is an urgent and unmet need for information on treatment and prognosis, although limited sources are available. The most common source of information are case reports and small series. These are seen as anecdotal evidence, and as such, are considered the lowest level of evidence in the hierarchy of evidence-based medicine [20]. Generalizability is limited due to the focus on single patients, with the inherent problems with methodology and statistics. Recently published guidelines [21] try to improve the quality of case reports. Indeed, case reports can provide useful insights into rare encounters into everyday practice [22, 23] and many provide a summary of the published literature on the subject, although these reviews are usually not performed according to the systematic strategies.

In order to investigate the value of case series, we performed an exhaustive systematic review of the literature and compared the data with a national cohort study, using record linkage between the national pathology database and the Netherlands cancer registry (NCR). Our first aim was to determine the value of case reports for the rare oncological scenarios, to determine whether this type of information is useful for clinical decision making, providing adequate and representative information. Our second aim was to summarize and analyse all available data on histologically proven colorectal metastases of breast cancer, in order to provide evidence for future treatment.

## Methods

### Strategy for search of articles and selection criteria

A comprehensive literature search for published studies was performed using Pubmed and Medline databases from inception to October 29th 2019, using the following terms "Colorectal Neoplasms/secondary "[MeSH] and “Rectal Neoplasms/secondary”[MeSH]. Additional searches were performed by manual cross-referencing. To avoid biases, there was no language restriction, nor was there selection on the basis of the number of included patients. We included all case reports and case series with original data that describe individual patients with secondary colorectal cancers that were derived from primary breast cancers. We excluded autopsy series, to limit ourselves to clinical useful scenarios. We further excluded studies describing primary colorectal cancer, metastases in small bowel and stomach, animal and in vitro studies, and studies describing direct invasion from other organs. Finally, all cases with metastases from other origins were excluded. Our strategy was registered in PROSPERO under number CRD42020149611.

### Strategy for population-based cohort study

Cases were identified in the Nationwide Dutch pathology databank (PALGA) between 1991 and 2019, and recorded under number lzv2019-144. PALGA has nationwide coverage since 1991 [24]. Selection was based on the following terms: “colon” (SNOMED term T67___) or “rectum” (SNOMED term T68___) or “bowel” (SNOMED term T50100) in combination with “metastasis”, where one of the first three terms should be the identifier of the target organ to exclude metastases derived from colorectal cancer. We then excluded all cases where the primary tumour was not breast cancer. Additional pathology reports of the selected patients were retrieved to determine the presence of histologically proven metastases in other organs and to retrieve details on the primary breast cancer. Data on endoscopic appearance and clinical symptoms were retrieved from the clinical data in pathology requests. Linkage of the patients with the NCR (registered under number K20.038) was performed to retrieve data on treatment of the primary tumour, metastasis and survival. This study was exempt from ethical approval because of anonymous data. Our study was conducted in compliance with national guidelines such as the Code of Conduct for the Use of Data in Health Research as issued by the Foundation Federation of Dutch Medical Scientific Societies (Federa), as well as the institutional regulations of PALGA and the NCR. All data were handled according to the General Data Protection Regulation. Consent for the design, data abstraction process from the NCR and storage protocols was obtained from the supervisory committee of the NCR. Scientific approval was obtained from the PALGA and the NCR scientific boards.

### Data extraction

For both the literature study and the population study we extracted patient data (age at time of breast cancer, sex), data on the primary breast cancer (location, type, size, nodal status, hormone receptor status, HER2 status), treatment of the breast cancer ((neo-)adjuvant systemic treatment, radiation therapy, surgical procedures), interval between diagnosis of primary breast cancer and diagnosis of colorectal metastases, symptoms, endoscopic presentation, surgical treatment of the colorectal metastases, presence of other metastases and survival since detection of the colorectal metastases. Data were entered in SPSS (SPSS for Windows, IBM SPSS Statistics 20, SPSS Inc, Chicago, Illinois, USA).

### Statistical analysis

All cases identified in our systematic literature search were considered and analysed as individual patients. The χ^2^-square test was used to compare demographics, tumour and treatment characteristics between groups. All tests of significance were two-tailed: differences at *P* values of less than 0.05 were considered to be significant. In survival analyses, overall survival (OS) was defined as the interval between the date of colonic metastasis until the date of death or until last follow-up. Patients who were alive at the end of follow-up were censored in survival analyses. OS curves were generated according to the Kaplan–Meier method. Comparison was made per cohort and based on the interval between primary breast cancer and diagnosis of colorectal metastases.

Factors influencing interval to colonic metastasis and survival were investigated with multivariate Cox regression analysis. For multivariate analysis the forward stepwise approach was applied. Statistical analyses were performed with the statistical software package SPSS.

## Results

### Systematic literature search results

**Fig. 1:**
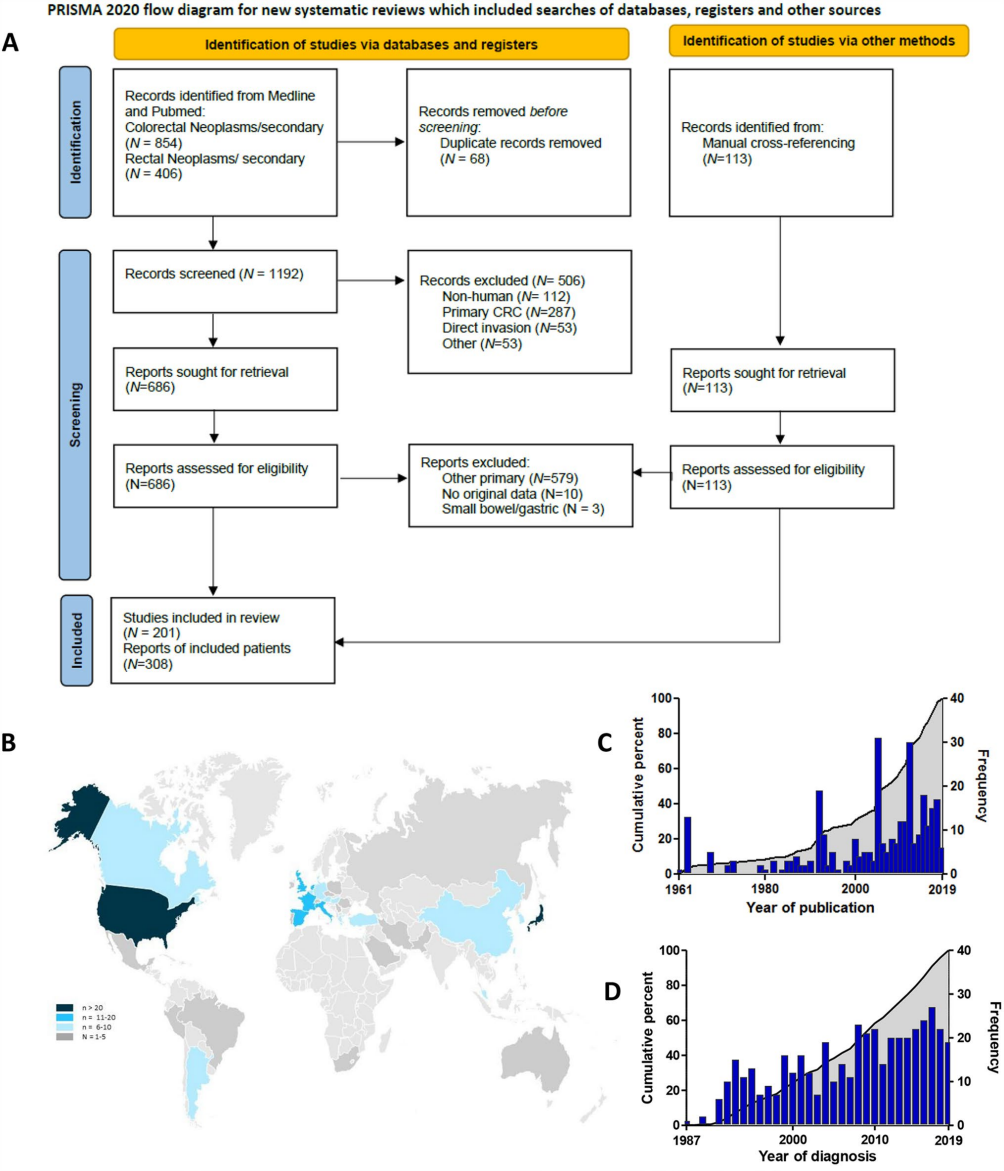
Information on the included cohorts. **A** Prisma flow chart of the systematic literature review, **B** Global distribution of cases derived from the systematic literature review. **C** Distribution of cases of the literature review over time. **D** Distribution of cases of real-life cohort over time (based on the year of diagnosis of colorectal metastasis)

A total of 406 studies were retrieved from PubMed and Medline for secondary rectal neoplasms and 854 were found using secondary colonic neoplasms as retrieval term. Duplicates were excluded (*N* = 68). A further 1086 studies were excluded because they did not meet general inclusion criteria (Fig. 1A) [25]. Manual cross-referencing resulted in an additional 113 case reports. Based on titles and abstracts (if available) 220 papers were included in our study. We excluded 10 studies since they did not have original data and three studies because the metastases were located in the small bowel or stomach rather than in the large bowel. The remaining 201 studies, which comprised 308 patients, were included in our individual case analysis (all references are presented in supplemental Table 1). Most studies (*N* = 187) described one patient each. Global distribution is depicted in Fig. 1B, with most patients from the USA (*N* = 100) and Japan (*N* = 32). One study (*N* = 35) was from an international collaboration [10]. The cases were described in the period between 1961 and 2019 (Fig. 1C).

Out of the 207 case reports and case series, 15 claimed a review of literature in the title (supplemental file). Of those, only six provided a table with individual patient data that varied from 6 to 39 cases, with variable information about symptoms, treatment, cancer type and interval. Another 8 papers showed similar tables, including 9–75 patients. Due to the limited information content, no firm conclusions could be drawn.

### Clinical and pathological data of the primary breast cancer from the literature cohort

Clinical and pathological data are summarized in Table 1. Most included patients were female (98.9%); in 37 cases, the sex was not explicitly stated in the case report. The age of patients at the time of diagnosis of the primary tumour varied between 23 and 84 years (mean age 57.3 years). The majority of patients presented with invasive lobular carcinoma (68.9%) versus invasive ductal carcinoma (26.6%). In six cases, mixed carcinoma was reported. More rare types were apocrine carcinoma (*N* = 2), metaplastic carcinoma (*N* = 2), mucinous carcinoma (*N* = 2), and papillary carcinoma (*N* = 1).There were many missing data from cases in the literature search, in 225 cases there was no information about HER2 status. Either size or T classification was not reported in 220 cases. Information on treatment of the primary breast cancer was also incomplete.

**Table 1:** Patient characteristics, characteristics of primary breast cancer and colonic metastases, per cohort

		Literature data	Real-life cohort	*P*-value
	Age			
	Mean (range)	57.2 (23–84)	57.8 (33–87)	0.56
	Sex			
	Female:male	268:3	451:3	0.52
Primary breast cancer	Location			
	Right breast	70 (43.5%)	172 (41.6%)	0.44
	Left breast	79 (49.1%)	220 (53.3%)	
	Bilateral cancer	12 (7.5%)	21 (5.1%)	
	Unknown	147	41	
	Tumor type			
	IDC	72 (26.6%)	175 (40.0%)	0.001
	ILC	186 (68.6%)	238 (54.3%)	
	Other	13 (4.8%)	25 (5.7%)	
	Missing	37	16	
	T category			
	T1	22 (24.7%)	189 (48.0%)	< 0.001
	T2	38 (43.2%)	146 (37.1%)	
	T3	18 (20.5%)	41 (10.4%)	
	T4	10 (11.4%)	18 (4.6%)	
	Unknown	220	60	
	N category			
	N0	42 (37.2%)	118 (29.0%)	< 0.001
	N1	26 (23.0%)	212 (52.1%)	
	N2/3	45 (39.8%)	77 (18.9%)	
	Unknown	195	47	
	M category			
	M0	140 (74.5%)	315 (73.3%)	0.75
	M1	48 (25.5%)	115 (26.7%)	
	Unknown	121	24	
	ER status			
	Positive	128 (88.3%)	323 (92.0%)	0.19
	Negative	17 (11.7%)	28 (8.0%)	
	Unknown	164	103	
	PR status			
	Positive	78 (67.8%)	223 (68.6%)	0.88
	Negative	37 (32.2%)	102 (31.4%)	
	Unknown	194	129	
	HER2 status			
	Positive	11 (13.1%)	16 (6.6%)	0.06
	Negative	72 (86.9%)	228 (93.4%)	
	Unknown	225	210	
	Surgery			< 0.001
	Local excision	24 (13.0%)	107 (23.9%)	
	Mastectomy	134 (72.8%)	243 (54.4%)	
	No breast surgery	26 (14.1%)	97 (21.7%)	
	Unknown	124	7	
	Lymph node surgery	140 (81.9%)	308 (68.9%)	0.001
	No lymph node surgery	31 (18.1%)	139 (31.1%)	
	Unknown	138	7	
	Radiotherapy			< 0.001
	Yes	65 (40.1%)	211 (56.3%)	
	No	97 (59.9%)	164 (43.7%)	
	Unknown	109	79	
	Chemotherapy			< 0.001
	Yes	97 (58.8%)	137 (36.5%)	
	No	68 (41.2%)	238 (63.5%)	
	Unknown	106	79	
	Hormonal therapy			0.43
	Yes	92 (55.4%)	222 (59.0%)	
	No	74 (44.6%)	154 (41.0%)	
	Unknown	105	78	
Colonic metastasis	Location			0.10
	Right colon	70 (33.0%)	136 (34.1%)	
	Left colon	43 (20.3%)	101 (25.3%)	
	Rectum	68 (32.1%)	93 (23.3%)	
	Multiple locations	31 (14.6%)	69 (17.3%)	
	Unknown	59	55	
	Macroscopy			0.008
	Normal	3 (1.5%)	–	
	Ulcus	10 (5.1%)	11 (3.4%)	
	Polyp	12 (6.1%)	18 (5.6%)	
	Cancer	76 (38.8%)	114 (35.6%)	
	IBD	18 (9.2%)	27 (8.4%)	
	Stenosis	47 (24.0%)	113 (35.3%)	
	Polyposis	7 (3.6%)	4 (1.3%)	
	Linitis plastica	13 (6.6%)	8 (2.5%)	
	Other	10 (5.1%)	25 (7.8%)	
	Unknown	75	134	
	Symptoms			0.064
	None	26 (12.4%)	9 (11.3%)	
	Blood loss	28 (13.3%)	5 (6.3%)	
	Diarrhea	47 (22.4%)	13 (16.3%)	
	Obstruction	72 (34.3%)	39 (48.8%)	
	Pain	29 (13.8%)	14 (17.5%)	
	Other	8 (3.2%)	-	
	Unknown	61	374	
	Surgery			< 0.001
	Yes	108 (52.7%)	138 (30.5%)	
	No	97 (47.3%)	314 (69.5%)	
	Unknown	65	2	

*IDC* invasive ductal carcinoma, *ILC* invasive lobular carcinoma, *T* tumor size, *N* nodal status, *M* metastasis, *ER* estrogen receptor, *PR* progesterone receptor

### Colorectal metastases from the literature cohort

Most metastases were present either in the right colon or the rectum (Table 1). The macroscopy of the metastases was highly variable, stenosis and ulcerating cancerous masses were more frequent than more specific patterns such as linitis plastica, polyposis and inflammatory bowel disease-like mucosal changes. The majority of patients presented with gastrointestinal complaints (obstruction 34.3%, diarrhoea 22.4% or rectal blood loss 13.8%). Patients without complaints (*N* = 26) underwent coloscopy because of population screening programs or abnormal imaging as part of the response evaluation of breast cancer treatment.

**Fig. 2:**
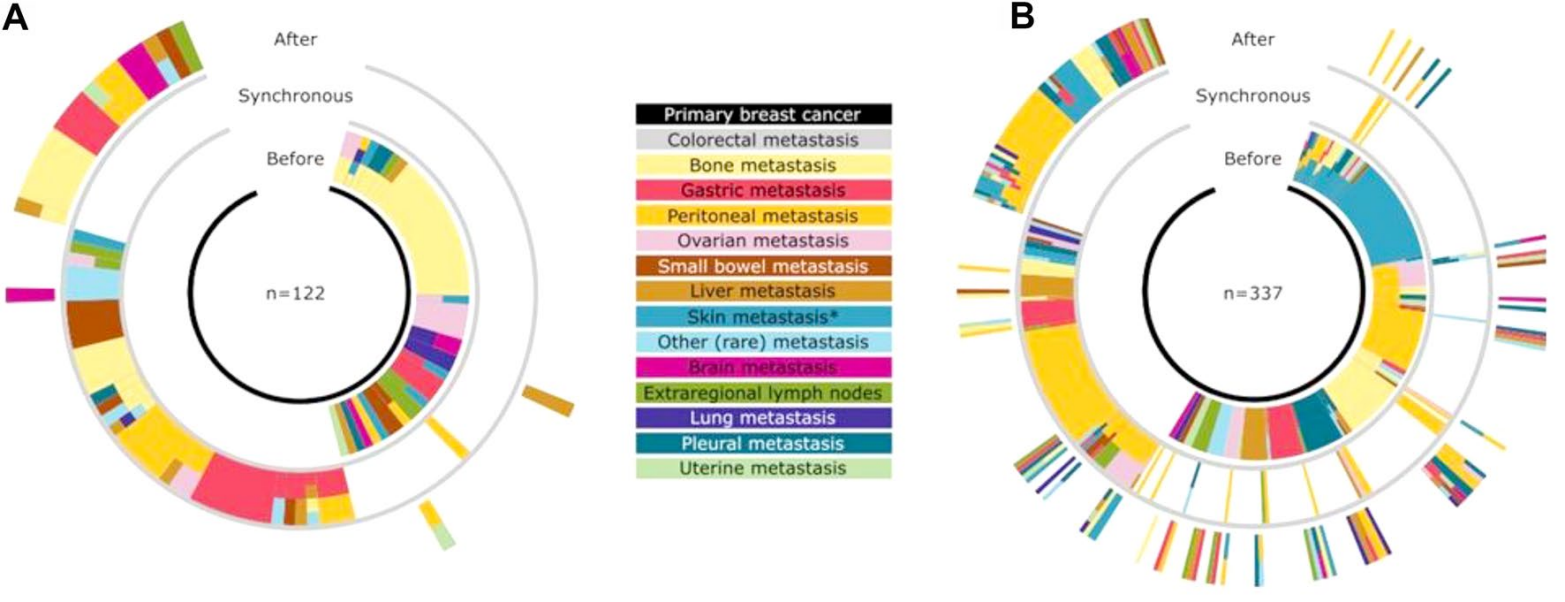
Distribution of other metastases in relation to the time of presentation of colorectal metastases in sunburst plots (**A** literature cohort, **B** real-life cohort). Reading the charts from the middle outwards, the different metastatic locations per patient are depicted in relation to the timing of the colorectal metastasis

Thirty-seven patients (17.9%) presented with colorectal metastasis as the first sign of breast cancer. The interval between the primary breast cancer and colorectal metastasis was as long as 300–500 months in some cases, with a median interval of 72 months for metachronous metastases (Fig. 2A). For 131 patients (42.5%), the colorectal metastasis was the first sign of metastatic disease, in 54 patients subsequent metastases were documented (Fig. 2C).

### Real-life cohort

A total of 10,735 patients were identified with our search strategy in the national pathology database between 1991 and 2019. The selection on colorectal breast cancer metastases resulted in 519 patients. Subsequently, we excluded all cases with only serosal involvement (these were considered peritoneal metastases), uncertainty about the primary tumour and those diagnosed at autopsy, as well as those that were coded incorrectly. This resulted in 454 patients. Based on the origin of the case reports and the national coverage of the population database, there is an overlap of 19 cases with the literature cohort. Linkage with the NCR was possible in 83% of cases, so full data including treatment was available for a total number of 375 cases. Linkage was not possible for cases diagnosed before 1991 (*N* = 72). In an additional 7 cases the cause of non-linkage was unknown. The distribution of colonic metastases in the real-life cohort over time is depicted in Fig. 1D.

### Clinical and pathological data of the primary breast cancer from the real-life cohort

Clinical and pathological data are summarized in Table 1. Most included patients were female (99.3%). The age of patients at the time of diagnosis of the primary breast tumour varied between 33 and 87 years (mean age 57.6 years). The majority of patients presented with invasive lobular carcinoma (53.3%) versus invasive ductal carcinoma (40.0%). In twenty cases, mixed carcinoma was reported. More rare types were medullary carcinoma (*N* = 1), neuroendocrine carcinoma (*N* = 1), mucinous carcinoma (*N* = 2), and papillary carcinoma (*N* = 1). The frequency of missing data from cases in the population search was limited, in 210 cases there was no information about HER2 status, which might be explained by the more recent introduction of this biomarker.

### Colorectal metastases from the population search

Most metastases were present in the right colon (34.3%; Table 1), the distribution was similar to the distribution from the case reports (*P* = 0.10). The macroscopic presentation of the metastasis differed slightly from the case reports (*P* = 0.051), mainly due to the classification of stenosis versus cancerous growth. Taken together, the occurrence of stenosis and cancerous growth were similar. The cases classified as other in the population study, were mostly described as oedema (*N* = 10), perforation (*N* = 4) or diverticulitis (*N* = 7). Symptoms, although rarely reported in the clinical information, were not different from the literature cohort.

**Fig. 3:**
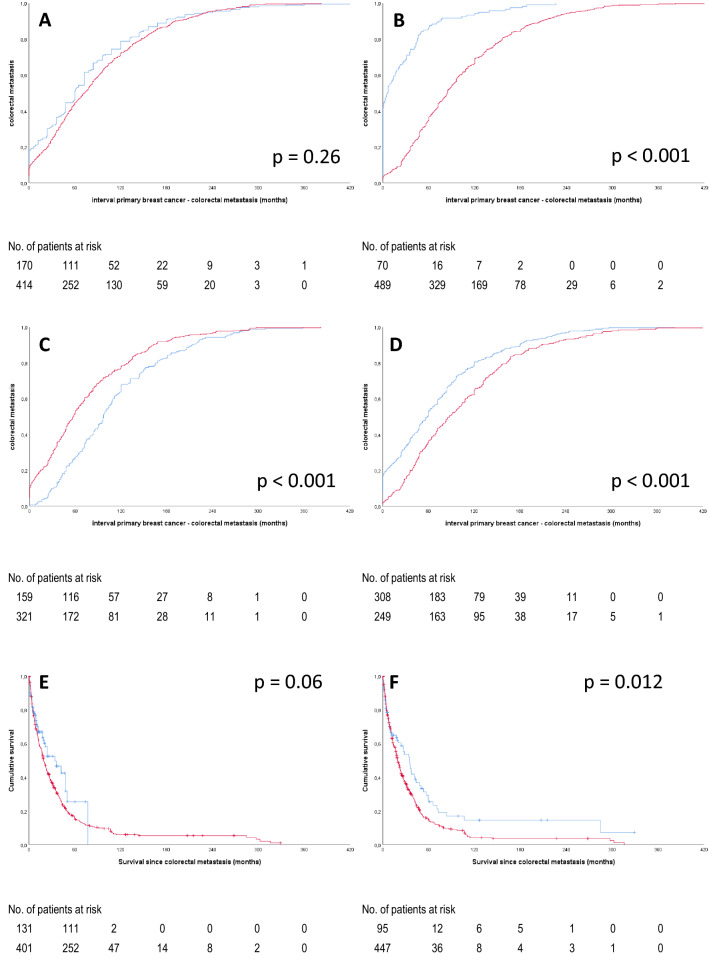
**A**–**D** Interval between primary breast cancer and diagnosis of colorectal metastasis. **A** Per cohort, in blue literature cohort, in red real-life cohort. **B** Impact of local surgery for breast cancer (blue: no surgery, red: surgery), **C** Impact of nodal status (red: no nodal metastases, blue: lymph node metastases), **D** Colorectal metastases as a first event (blue). In red colorectal metastases as a subsequent event. **E**, **F** Overall survival after development of colorectal metastases. **E** Overall survival per cohort (blue: literature cohort, red: population cohort), **F** Overall survival depending on interval between primary breast cancer and diagnosis of colorectal metastases (blue: within 1 year, red: interval is over 1 year). (Color figure online)

Twenty-nine patients (6.7%) presented with colorectal metastases as the first sign of breast cancer. The interval between the primary breast cancer and colorectal metastasis (Fig. 3A) was over 300 months for three patients, with a median interval of 79 months (mean interval 95 months), which is comparable to the data of the literature search. For 244 patients, the colorectal metastases were the first histological proven metastasis (47.0%), in 134 patients subsequent metastases were documented (Fig. 2A).

### Comparison of cohorts

Both cohorts were comparable in age, gender, localization, hormone receptor status and hormonal therapy use distribution (Table 1). In the real-life cohort more invasive ductal carcinomas were present (40.0% vs. 26.6%, *P* = 0.001) and the tumours were smaller (48.0% pT1 vs. 24.7%, *P* < 0.001), but more often presented with lymph node metastases (71% vs. 62.8%, *P* < 0.001). In the real-life cohort, we observed less mastectomies (54.4% vs. 72.8%, *P* < 0.001), less axillary lymph node dissections (68.9% vs. 81.9%, *P* = 0.001), more radiotherapy (56.3% vs. 40.1%, *P* = 0.001) and less chemotherapy (36.5% vs. 58.8%, *P* < 0.001). There was no difference in the percentage of patients treated with hormonal therapy. Resection of the metastases was performed more frequently in patients from the literature cohort, 52.7% vs. 30.5% (*P* < 0.001), respectively.

### Comparison of outcome data

Data for outcome were available for 208 patients from the literature cohort and 451 patients from the population cohort. The median time to development of the colorectal metastasis from the diagnosis of breast cancer was 68.0 months (95% confidence interval (CI) 61.4–74.6) and similar in both cohorts (Table 2, Fig. 3A). In the literature cohort, there were more patients with colorectal metastases as the first presentation (18% vs. 7%, *P* < 0.001). Four factors influenced the timing of colorectal metastases (Table 2), in particular the presence of synchronous metastases and other metastatic sites before the development of colonic metastasis (Fig. 3D). There was no effect of subtype or biomarkers on the timing of colorectal metastases, nor an effect on survival. For patients with other synchronous metastases the interval to detection of colorectal metastases was 36 months (95% CI 27–45 months, patients with synchronous colorectal metastases excluded) versus 85 months (95%CI 78–92 months) in patients without synchronous metastases (*P* < 0.001), suggesting that colorectal metastases are a late event. Furthermore, surgery of the primary tumour (Table 2, Fig. 3B) and nodal status (Fig. 3C) were related to the length of this interval. In a multivariate approach, all these factors, including cohort, were significant factors in the timing of metastases.

**Table 2:** Cox regression for time to development of colorectal metastasis (univariate and multivariate) and for survival after colorectal metastasis

	Time to development of colorectal metastasis			Survival after colorectal metastasis	
	*N*	Univariate (HR, 95%CI)	Multivariate (HR, 95%CI)	*N*	Univariate (HR, 95% CI)
Cohort					
Literature	208	1.1 (0.9–1.3)	1.4 (1.1–1.7)	132	1.0
Population	451	1.0	1.0	454	0.8 (0.6–1.0)
Surgery primary tumor					
Yes	506	1.0	1.0	445	1.0
No	121	3.6 (2.9–4.5)	4.3 (3.5–5.5)	116	1.0 (0.7–1.2)
Location primary tumor					
Left	298	0.8 (0.6–1.2)		215	1.2 (0.7–1.9)
Right	241	0.7 (0.5–1.0)		268	1.2 (0.7–1.9)
Bilateral	32	1.0		26	1.0
Histologic subtype					
IDC	227	0.9 (0.6–1.3)		203	0.7 (0.5–1.1)
ILC	370	1.1 (0.8–1.5)		324	0.8 (0.5–1.2)
Other	36	1.0		34	1.0
Molecular subtype					
ER + /HER2 – or unknown	260	0.9 (0.6–1.4)		242	0.7 (0.4–1.2)
ER + /HER2 +	22	1.0 (0.6–2.0)		18	0.5 (0.2–1.1)
Triple negative	24	1.0		20	1.0
Size of primary tumor					
pT1	211	0.8 (0.6–1.3)		202	1.0 (0.6–1.7)
pT2	183	0.7 (0.4–1.0)		169	1.2 (0.7–2.0)
pT3	59	0.8 (0.5–1.3)		53	0.9 (0.5–1.6)
pT4	29	1.0		23	1.0
Nodal status					
pN0	160	0.7 (0.5–0.8)	0.8 (0.6–1.0)	138	1.0 (0.8–1.2)
pN +	358	1.0	1.0	335	1.0
Synchronous metastases					
Yes	158	0.3 (0.2–0.4)	0.3 (0.2–0.5)	143	1.0
No	452	1.0	1.0	398	1.1 (0.9–1.4)
Colonic metastases as first event					
Yes	371	1.4 (1.3–1.7)	1.5 (1.3–1.8)	371	0.9 (0.7–1.1)
No	254	1.0	1.0	254	1.0
Time to development of colorectal metastasis	–	–	–		
< 1 year				95	0.7 (0.5–0.9)
> 1 year				488	1
Location metastasis	–	–	–		
Right				182	1.0 (0.7–1.3)
Left				120	1.1 (0.8–1.5)
Rectum				138	1.0 (0.7–1.4)
Multiple				85	1
Resection metastasis	–	–	–		
Yes				211	1
No				365	1.1 (0.9–1.4)

We repeated the analyses for stage I-III breast cancer to exclude the effects of treatment decisions based on the presence of other metastases. The same factors were identified here: colorectal metastases as the first event (hazard ratio (HR) 1.4 (95% CI 1.2–1.7)), surgery of the primary tumour (HR 5.1, 95%CI 1.2–20.7)) and nodal status (HR 0.8 (95%CI 0.7–1.0)).

The median overall survival of all patients after development of colorectal metastases was 20.6 months (95% CI 18.0–23.1). In Table 2 the factors that might influence overall survival after colorectal metastases are summarized. Neither tumour characteristics nor metastasis-associated characteristics showed a significant relation with outcome. Only the length of the interval between the primary tumour and the colorectal metastases influenced survival, with a shorter interval paradoxically associated with longer survival (median survival 35 vs. 19.3 months, *P* = 0.012). Patients with synchronous colorectal metastases (*N* = 59) did have a median overall survival of 35.9 months (95% CI 20.2–51.6 months). In the real-life cohort overall survival was shorter than in the literature cohort (median survival 19.3 vs. 34.0 months, HR 0.8, 95% CI 0.6–1.0), albeit not significant (*P* = 0.06). When analysed in the cohort without synchronous metastases this difference was more evident (HR 0.5 (95%CI 0.3–0.9)).

## Discussion

We have investigated the largest series of breast cancer patients with colorectal metastases ever published in literature and describe the presenting symptoms and morphological patterns of this unusual localization of breast cancer metastases. Also, we have shown that the median overall survival after development of the colorectal metastases varies from 19 months for the real-life cohort to 34 months for the literature cohort. While we were able to identify several factors that were related with the interval between the primary tumour and the colorectal metastasis, the only factor that influences overall survival since colorectal metastasis was the length of this interval.

In care for oncology patients the boundaries of treatment are continuously sought for. It is incontrovertible that metastatic disease is no longer observed as an endpoint of disease in which treatment with curative intent is no longer possible. Multidisciplinary teams even treat patients with several metastases, thereby continuously outweighing the potential benefits over possible harm. It is, however, due to the shifting paradigm difficult to estimate the potential benefit of local treatments in this patient category. Obviously, randomized trials or even cohort studies are missing, although a substantiated estimation of prognosis is desirable. In the current study we applied two different strategies to study outcome and effects of therapy in a rare oncologic condition: colorectal metastases of breast cancer.

We have collected the largest series of patients with breast cancer and subsequent development of colorectal metastases. Two separate cohorts, one individual patient data meta-analysis based on a systematic review of case reports and small cases series and a real-life cohort resulted in a total number of 762 patients with 67 months as the median time to development of colorectal metastasis. Paradoxically, patients that presented within 12 months with colorectal metastases had a significant longer survival time after development of colorectal metastases. In general, longer disease-free intervals are associated with longer survival [26].

For almost all cancers, colorectal metastases are rare and peritoneal metastases are more common. Therefore, it might be possible that part of our patients (both in the real life cohort and in the literature search) might have peritoneal metastases infiltrating the colon rather than intraluminal colorectal metastases. Each individual case was evaluated for this and we excluded cases without mucosal presence of metastatic disease. From the patients that presented with other metastases synchronous with the colorectal metastases, approximately 50% were indeed peritoneal in origin (data not shown).Several differences were observed between both cohorts, for which theoretically multiple causes could be identified, including differences in the year of diagnosis or the country of origin. These causes are likely to contribute to the observed differences in outcomes. A sensitivity analysis for the timing of diagnosis of the primary tumour, where we arbitrarily included all papers with a publication date after 1995 did not show any impact on the observed differences between the cohorts for type, stage and treatment (data not shown). An additional sensitivity analysis comparing the Dutch cases from the literature review (*N* = 19) with the entire population cohort was hampered by the lack of data. Tumour type was the only evaluable factor with significantly less IDC in the literature cohort than in the real-life cohort (26.6% versus 40%, *P* = 0.015). Relatively large numbers were available from Japan (*N* = 39) and the USA (*N* = 100). However, due to limited follow-up data it was not possible to establish a potential effect of the country of origin on outcomes.

There are several limitations inherent to individual patient data meta-analyses, that are illustrated by the comparison with the real-life cohort. First, case reports are subject to selection bias and publication bias. In general, case reports represent rare or uncommon clinical situations, possibly leading to an overestimation of the frequency of these clinical situations. This also accounts for the reporting of corresponding outcomes, which can be on either side of the spectrum (i.e. ranging from extremely poor prognosis to surprisingly long survival). Surprisingly, there was no difference in the number of male breast cancer patients.

Second, not all data regarding the primary tumour and treatment characteristics was available for each individual patient (summarized in supplemental Fig. 1). Occasionally, the time course of the disease could not be reconstructed. This is reflected by different patient numbers in patient characteristics and survival analyses. The real-life cohort was lacking in sufficient data on symptoms that accompanied the colorectal metastases, this was to be expected, since these cases were retrieved from the pathology database and the cancer registry, that do not systematically collect these data.

In this study we have shown that, despite the obvious differences between case reports and large population-based studies, the information that can be retrieved from a systematic review of cases in the literature is valuable and comparable to real-life cohorts. Thus, this is a valuable tool to study rare oncological scenarios. However, a systematic approach is essential to retrieve complete and extensive information. Currently, literature reviews that are present in association with case reports are incomplete and highly variable in informational content.

For breast cancer patients, we have identified symptoms, endoscopic variety and clinicopathological characterization of colorectal metastases. More important, we have shown that this type of metastasis generally occurs long after the diagnosis of the primary tumour, but that this diagnosis is not generally accompanied by an immediate infaust outcome. Although surgical resection does not contribute to better outcome, it might be considered for treatment of local symptoms.

## Supplementary Information

Below is the link to the electronic supplementary material.

Supplementary file1 (DOCX 187 kb)—Completeness of the variables per cohort.

Supplementary file2 (DOCX 129 kb)—Reference list of all included case reports.

## Data Availability

The data underlying this article were provided by PALGA and NCR by permission. Data will be shared on reasonable request to the corresponding author with permission from PALGA and NCR.

## Abbreviations

CI: Confidence interval
ER: Estrogen receptor
HR: Hazard ratio
IDC: Invasive ductal carcinoma
ILC: Invasive lobular carcinoma
NCR: Netherlands cancer registry
PR: Progesterone receptor
OS: Overall survival

## References

[1] Caswell-Jin JL, Plevritis SK, Tian L, Cadham CJ, Xu C, Stout NK, Sledge GW, Mandelblatt JS, Kurian AW (2018). Change in survival in metastatic breast cancer with treatment advances: meta-analysis and systematic review. JNCI Cancer Spectr.

[2] Brouwer NPM, Bos A, Lemmens V, Tanis PJ, Hugen N, Nagtegaal ID, de Wilt JHW, Verhoeven RHA (2018). An overview of 25 years of incidence, treatment and outcome of colorectal cancer patients. Int J Cancer.

[3] Sundermeyer ML, Meropol NJ, Rogatko A, Wang H, Cohen SJ (2005). Changing patterns of bone and brain metastases in patients with colorectal cancer. Clin Colorectal Cancer.

[4] Van Cutsem E, Cervantes A, Adam R, Sobrero A, Van Krieken JH, Aderka D, Aranda Aguilar E, Bardelli A, Benson A, Bodoky G (2016). ESMO consensus guidelines for the management of patients with metastatic colorectal cancer. Ann Oncol.

[5] Chiorean EG, Nandakumar G, Fadelu T, Temin S, Alarcon-Rozas AE, Bejarano S, Croitoru AE, Grover S, Lohar PV, Odhiambo A (2020). Treatment of patients with late-stage colorectal cancer: ASCO resource-stratified guideline. JCO Glob Oncol.

[6] Van Poznak C, Somerfield MR, Barlow WE, Biermann JS, Bosserman LD, Clemons MJ, Dhesy-Thind SK, Dillmon MS, Eisen A, Frank ES (2017). Role of bone-modifying agents in metastatic breast cancer: an American society of clinical oncology-cancer care ontario focused guideline update. J Clin Oncol.

[7] Terada T (2013). Histopathologic study of the rectum in 1464 consecutive rectal specimens in a single Japanese hospital: II malignant lesions. Int J Clin Exp Pathol.

[8] Logan RF, Patnick J, Nickerson C, Coleman L, Rutter MD, von Wagner C (2012). English bowel cancer screening evaluation C: outcomes of the bowel cancer screening programme (BCSP) in England after the first 1 million tests. Gut.

[9] Terada T (2012). Pathologic observations of the duodenum in 615 consecutive duodenal specimens in a single Japanese hospital: II malignant lesions. Int J Clin Exp Pathol.

[10] Mourra N, Jouret-Mourin A, Lazure T, Audard V, Albiges L, Malbois M, Bouzourene H, Duvillard P (2012). Metastatic tumors to the colon and rectum: a multi-institutional study. Arch Pathol Lab Med.

[11] Berge T, Lundberg S (1977). Cancer in Malmo 1958–1969. An autopsy study. Acta Pathol Microbiol Scand Suppl.

[12] Sung H, Ferlay J, Siegel RL, Laversanne M, Soerjomataram I, Jemal A, Bray F (2021). Global cancer statistics 2020: GLOBOCAN estimates of incidence and mortality worldwide for 36 cancers in 185 countries. CA Cancer J Clin.

[13] van Maaren MC, de Munck L, Strobbe LJA, Sonke GS, Westenend PJ, Smidt ML, Poortmans PMP, Siesling S (2019). Ten-year recurrence rates for breast cancer subtypes in the Netherlands: a large population-based study. Int J Cancer.

[14] Vondeling GT, Menezes GL, Dvortsin EP, Jansman FGA, Konings IR, Postma MJ, Rozenbaum MH (2018). Burden of early, advanced and metastatic breast cancer in The Netherlands. BMC Cancer.

[15] Cummings MC, Simpson PT, Reid LE, Jayanthan J, Skerman J, Song S, McCart Reed AE, Kutasovic JR, Morey AL, Marquart L (2014). Metastatic progression of breast cancer: insights from 50 years of autopsies. J Pathol.

[16] Borst MJ, Ingold JA (1993). Metastatic patterns of invasive lobular versus invasive ductal carcinoma of the breast. Surgery.

[17] Harris M, Howell A, Chrissohou M, Swindell RI, Hudson M, Sellwood RA (1984). A comparison of the metastatic pattern of infiltrating lobular carcinoma and infiltrating duct carcinoma of the breast. Br J Cancer.

[18] Clavien PA, Laffer U, Torhost J, Harder F (1990). Gastro-intestinal metastases as first clinical manifestation of the dissemination of a breast cancer. Eur J Surg Oncol.

[19] Cifuentes N, Pickren JW (1979). Metastases from carcinoma of mammary gland: an autopsy study. J Surg Oncol.

[20] Sackett DL, Rosenberg WM, Gray JA, Haynes RB, Richardson WS (1996). Evidence based medicine: what it is and what it isn't. BMJ.

[21] Riley DS, Barber MS, Kienle GS, Aronson JK, von Schoen-Angerer T, Tugwell P, Kiene H, Helfand M, Altman DG, Sox H (2017). CARE guidelines for case reports: explanation and elaboration document. J Clin Epidemiol.

[22] Yitschaky O, Yitschaky M, Zadik Y (2011). Case report on trial: Do you, Doctor, swear to tell the truth, the whole truth and nothing but the truth?. J Med Case Rep.

[23] McWhinney IR (2001). The value of case studies. Eur J General Practice.

[24] Casparie M, Tiebosch AT, Burger G, Blauwgeers H, van de Pol A, van Krieken JH, Meijer GA (2007). Pathology databanking and biobanking in The Netherlands, a central role for PALGA, the nationwide histopathology and cytopathology data network and archive. Cell Oncol.

[25] Page MJ, McKenzie JE, Bossuyt PM, Boutron I, Hoffmann TC, Mulrow CD, Shamseer L, Tetzlaff JM, Akl EA, Brennan SE (2021). The PRISMA 2020 statement: an updated guideline for reporting systematic reviews. BMJ.

[26] Witteveen A, Kwast AB, Sonke GS, Ij MJ, Siesling S (2015). Survival after locoregional recurrence or second primary breast cancer: impact of the disease-free interval. PLoS ONE.

