# Supplementary material for: The value of case reports in rare oncological scenarios: mixed method analysis of colorectal metastases from breast cancer
Source: Clin Exp Metastasis. 2023 Apr 27;40(3):205–16. doi: 10.1007/s10585-023-10207-9 (PMC10232590; doi:10.1007/s10585-023-10207-9)

**Supplemental file**

List of case reports and case series [1-201]

1. Abid A, Moffa C, Monga DK. Breast cancer metastasis to the GI tract may mimic primary gastric cancer. J Clin Oncol 2013; 31: e106-107.

2. Aihara H, Drage MG, Agoston A, Thompson CC. Metastatic Breast Cancer Mimicking a Solitary Small Colonic Erosion. Am J Gastroenterol 2017; 112: 677.

3. Albero-Gonzalez RG-B, J.; Vazquez-de lsa Heras, I.; Martinez-Orfila, J.; Seoane-Urgorri, A.; Naranjo-Hans, D.; Corominas, J.M.; Iglesias-Coma, M.; Alameda-Quitllet, F. Lobular breast carcinoma with colonic metastases: a synchornous diagnosis in a 4-day period. Human Pathol: case reports 2017; 7: 27-30.

4. Ali WM, Z.K.; Thekkinkattil, D. Colonic metastasis from a breast carcinoma, an unusual colonoscopic finding. BJMP 2016; 9: a901.

5. Al-Qahtani MS. Gut metastasis from breast carcinoma. Saudi Med J 2007; 28: 1590-1592.

6. Alves de Lima DC, Alberti LR. Breast cancer metastasis to the colon. Endoscopy 2011; 43 Suppl 2 UCTN: E143-144.

7. Ambroggi M, Stroppa EM, Mordenti P et al. Metastatic breast cancer to the gastrointestinal tract: report of five cases and review of the literature. Int J Breast Cancer 2012; 2012: 439023.

8. Amin AA, Reddy A, Jha M, Prasad K. Rectal metastasis from breast cancer: an interval of 17 years. BMJ Case Rep 2011; 2011.

9. Andriola V, Piscitelli D, De Fazio M, Altomare DF. Massive colonic metastasis from breast cancer 23 years after mastectomy. Int J Colorectal Dis 2015; 30: 427-428.

10. Arrangoiz R, Papavasiliou P, Dushkin H, Farma JM. Case report and literature review: Metastatic lobular carcinoma of the breast an unusual presentation. Int J Surg Case Rep 2011; 2: 301-305.

11. Asch MJ, Wiedel PD, Habif DV. Gastrointestinal metastases from crcinoma of the breast. Autopsy study and 18 cases requiring operative intervention. Arch Surg 1968; 96: 840-843.

12. Babb RR, Trollope M. Colonic metastases from breast cancer. Surg Endosc 2001; 15: 530.

13. Bailey CM, Gilbert JM. Avoiding inappropriate surgery for secondary rectal cancer. Eur J Surg Oncol 2002; 28: 220-224.

14. Balakrishnan B, Shaik S, Burman-Solovyeva I. An Unusual Clinical Presentation of Gastrointestinal Metastasis From Invasive Lobular Carcinoma of Breast. J Investig Med High Impact Case Rep 2016; 4: 2324709616639723.

15. Balja MP, Vrdoljak DV, Stanec M et al. Rectal metastasis from lobular carcinoma of the breast: a case report. Coll Antropol 2010; 34: 719-721.

16. Balthazar EJ, Rosenberg HD, Davidian MM. Primary and metastatic scirrrhous carcinoma of the rectum. AJR Am J Roentgenol 1979; 132: 711-715.

17. Bamias A, Baltayiannis G, Kamina S et al. Rectal metastases from lobular carcinoma of the breast: report of a case and literature review. Ann Oncol 2001; 12: 715-718.

18. Bar-Zohar D, Kluger Y, Michowitz M. Breast cancer metastasizing to the rectum. Isr Med Assoc J 2001; 3: 624-625.

19. Basu S, Last A, Shinkfield M. Invasive lobular carcinoma of the breast presenting successively with colonic and gastric metastases. Int J Clin Pract 2002; 56: 623-625.

20. Birla R, Mahawar KK, Orizu M et al. Caecal metastasis from breast cancer presenting as intestinal obstruction. World J Surg Oncol 2008; 6: 47.

21. Blachman-Braun R, Felemovicius I, Barker K et al. Widespread metastatic breast cancer to the bowel: an unexpected finding during colonoscopy. Oxf Med Case Reports 2019; 2019: omy133.

22. Bognar G, Barabas L, Diczhazi C, Ondrejka P. [Large bowel obstruction due to colonic and omental metastasis as the first clinical sign of metastatic ductal carcinoma of the breast]. Magy Seb 2009; 62: 312-315.

23. Borst MJ, Ingold JA. Metastatic patterns of invasive lobular versus invasive ductal carcinoma of the breast. Surgery 1993; 114: 637-641; discussion 641-632.

24. Buka D, Dvorak J, Richter I et al. Gastric and Colorectal Metastases of Lobular Breast Carcinoma: A Case Report. Acta Medica (Hradec Kralove) 2016; 59: 18-21.

25. Burgesser MV, Calafat P, Diller A. [Colonic metastases of breast infiltrating lobular carcinoma: atypical presentation of a clinical case]. Rev Fac Cien Med Univ Nac Cordoba 2010; 67: 85-88.

26. Bustamante-Balen M, Navarro-Hervas M, Cuevas JM, Garcia-Diez JV. [Colon metastasis of a lobular breast carcinoma]. Rev Esp Enferm Dig 2008; 100: 249-250.

27. Calafat P, de Diller AB, Sanchez C. [Breast carcinoma metastasis in ileum-colon and gallbladder simulating inflammatory diseases]. Rev Fac Cien Med Univ Nac Cordoba 1999; 56: 123-127.

28. Cano-Maldonado AJ, Diaz-Tie M, Vives-Rodriguez E et al. [Rectal metastasis of lobular breast carcinoma]. Rev Esp Enferm Dig 2008; 100: 440-442.

29. Carcoforo PR, M.T.; Langan, R.C.; Lanzara, S.; Portinari, M.; Maestroni, U.; Palini, G.M.; Zanzi, M.V.; Bonazza, S.; Pedriali, M.; Feo, C.V.; Stojandinovic, A.; Aviral, I. Infiltrating lobular carcinoma of the breast presenting as gastrointestinal obstruction: a mini review. J Cancer 2012; 3: 328-332.

30. Carroll J, Vujcich E, Lambrianides AL. Metastatic lobular carcinoma of the breast: an unusual case. ANZ J Surg 2018; 88: 378-379.

31. Cervi G, Vettoretto N, Vinco A et al. Rectal localization of metastatic lobular breast cancer: report of a case. Dis Colon Rectum 2001; 44: 453-455.

32. Cestafe MCG, N.C.; Sanchez Refoyo, F.; Ayastuy, J.G.; Urizar, J.L. M.; Lozano, J.L.P.; Alustiza, J.E.; Ramos, J.D.S. [Breast cancer: ovarian and colonic metastasis]. Prog Obst Gin 2014; 57: 37-39.

33. Ceulemans R, Oyen R, Franssens Y, Ponette E. Intestinal submucosal metastases from breast cancer. J Belge Radiol 1994; 77: 209.

34. Cherian N, Qureshi NA, Cairncross C, Solkar M. Invasive lobular breast carcinoma metastasising to the rectum. BMJ Case Rep 2017; 2017.

35. Cherkes VL, Oskirko DA, Barsukov Iu A, Kuznetsov VV. [Rectal metastases of breast carcinoma]. Khirurgiia (Mosk) 2005; 68-69.

36. Clavien PA, Laffer U, Torhost J, Harder F. Gastro-intestinal metastases as first clinical manifestation of the dissemination of a breast cancer. Eur J Surg Oncol 1990; 16: 121-126.

37. Critchley AC, Harvey J, Carr M, Iwuchukwu O. Synchronous gastric and colonic metastases of invasive lobular breast carcinoma: case report and review of the literature. Ann R Coll Surg Engl 2011; 93: e49-50.

38. Daniels IR, Layer GT, Chisholm EM. Bowel obstruction due to extrinsic compression by metastatic lobular carcinoma of the breast. J R Soc Promot Health 2002; 122: 61-62.

39. Darcha C, Orliaguet T, Le Bouedec G et al. [Digestive system metastases from breast cancer. Report of two cases]. Ann Pathol 1993; 13: 250-252.

40. Defrawi T, Goyal A, Duan X et al. Breast cancer metastatic to the colon 20 years after bilateral mastectomy. Endoscopy 2006; 38 Suppl 2: E1.

41. Del Cimmuto P, De Lauretis D, Gentile G, Zerilli M. [Atypical metastases of breast carcinoma. An isolated localization in the transverse colon treated with radical surgery]. Minerva Chir 1982; 37: 869-873.

42. Dhar S, Kulaylat MN, Gordon K et al. Solitary papillary breast carcinoma metastasis to the large bowel presenting as primary colon carcinoma: case report and review of the literature. Am Surg 2003; 69: 799-803.

43. Doyle DJ, Relihan N, Redmond HP, Barry JE. Metastatic manifestations of invasive lobular breast carcinoma. Clin Radiol 2005; 60: 271-274.

44. Easter DW, Jamshidipour R, McQuaid K. Laparoscopy to correctly diagnose and stage metastatic breast cancer mimicking Crohn's disease. Surg Endosc 1995; 9: 820-823.

45. Ede C, Benn CA, Fearnhead K et al. Should all lobular breast carcinoma undergo staging gastrointestinal endoscopy? Breast J 2019; 25: 340-342.

46. Efthimiadis C, Kosmidis C, Fotiadis P et al. Breast cancer metastatic to the rectum: a case report. Tech Coloproctol 2011; 15 Suppl 1: S91-93.

47. Eljabu W, Finch G, Nottingham J, Vaingankar N. Metastatic deposits of breast lobular carcinoma to small bowel and rectum. Int J Breast Cancer 2011; 2011: 413949.

48. Elsaify W, Kanwar A, Nagarajan S. Lobular carcinoma of the breast: presentation, histopathological features and management of rectal metastasis. Breast J 2012; 18: 597-599.

49. Eyres KS, Sainsbury JR. Large bowel obstruction due to metastatic breast cancer: an unusual presentation of recurrent disease. Br J Clin Pract 1990; 44: 333-334.

50. Falco G, Mele S, Zizzo M et al. Colonic metastasis from breast carcinoma detection by CESM and PET/CT: A case report. Medicine (Baltimore) 2018; 97: e10888.

51. Fayemi AO, Ali M, Braun EV. Metastatic carcinoma simulating linitis plastica of the colon. A case report. Am J Gastroenterol 1979; 71: 311-314.

52. Feng CL, Chou JW, Huang SF. Colonic metastasis from carcinoma of the breast presenting with colonic erosion. Endoscopy 2009; 41 Suppl 2: E276-277.

53. Flamme F, Jacobowitz D, Feoli F et al. Diminutive polyp: a rare presentation of breast cancer metastases to the colon. Acta Gastroenterol Belg 1994; 57: 260-263.

54. Garret M, Devars du Mayne JF, Barge J et al. [Linitis plastica of the colon and stomach following breast cancer]. Gastroenterol Clin Biol 1986; 10: 276-277.

55. Gerova VA, Tankova LT, Mihova AA et al. Gastrointestinal metastases from breast cancer: report of two cases. Hepatogastroenterology 2012; 59: 178-181.

56. Gifaldi AS, Petros JG, Wolfe GR. Metastatic breast carcinoma presenting as persistent diarrhea. J Surg Oncol 1992; 51: 211-215.

57. Gilg MM, Grochenig HP, Schlemmer A et al. Secondary tumors of the GI tract: origin, histology, and endoscopic findings. Gastrointest Endosc 2018; 88: 151-158 e151.

58. Gizzi G, Santini D, Guido A, Fuccio L. Single colonic metastasis from breast cancer 11 years after mastectomy. BMJ Case Rep 2015; 2015.

59. Gleeson FC, Clain JE, Rajan E et al. Secondary linitis plastica of the rectum: EUS features and tissue diagnosis (with video). Gastrointest Endosc 2008; 68: 591-596.

60. Graham WPG, L. Gastro-intestinal metastases from carcinoma of the breast. Ann Surg 1964; 159: 477-480.

61. Grosdidier J, Boissel P, Bresler L, Froment N. [Rectal metastasis of breast cancer. Apropos of 2 cases]. J Chir (Paris) 1985; 122: 87-91.

62. Guo JJ, Yang DQ, Sun KK, Shen DH. [Rectal metastases from carcinoma of breast: report of a case]. Zhonghua Bing Li Xue Za Zhi 2009; 38: 492-493.

63. Guzman-Calderon E. Rectal Metastases from Breast Cancer. A Case Report. J Gastrointest Cancer 2017; 48: 205-207.

64. Hamish IH, Paran H, Cohen D, Gutman M. Metastatic breast cancer imitating acute diverticulitis. Isr Med Assoc J 2011; 13: 321-322.

65. Harslof SS, Andersen LM, Hoyer U, Christiansen JJ. [Breast cancer metastasis to the colon]. Ugeskr Laeger 2010; 172: 2309-2310.

66. Hasegawa SS, N.; Shino,K.; Higughi, A.; Nakayama, T.; Ike, H. . [A case of breast cancer with rectal metastasis diagnosed 17 years after breast surgery for which hormone therapy has been effective. Nihon Rinsho Geka Gakkai Zasshi 2013; 74: 2091-2095.

67. Haubrich WS. Adenocarcinoma of the breast metastatic to the rectum. Gastrointest Endosc 1985; 31: 403-404.

68. Hirano A, Nakamura S, Fujita K et al. Aphthous lesions of the colon as a manifestation of metastasized breast cancer. Endoscopy 2011; 43 Suppl 2 UCTN: E131-132.

69. Horimoto Y, Hirashima T, Arakawa A et al. Metastatic colonic and gastric polyps from breast cancer resembling hyperplastic polyps. Surg Case Rep 2018; 4: 23.

70. Hsieh PS, Yeh CY, Chen JR, Changchien CR. Ileocecal breast carcinoma metastasis. Int J Colorectal Dis 2004; 19: 607-608.

71. Ikeda A, Takahashi Y, Nakajima S et al. [A Case of Colon Metastasis from Breast Cancer with Resection by Laparoscopic Surgery]. Gan To Kagaku Ryoho 2016; 43: 1745-1747.

72. Ikeda Y, Morita N, Ikeda T. Metachronous rectal metastasis from invasive ductal carcinoma of the male breast. Endoscopy 2008; 40 Suppl 2: E108-109.

73. Jeong W, Trembath DG, Baron TH. Endoscopic ultrasound-guided fine needle biopsy through the interstices of a colonic stent for the diagnosis of metastatic breast cancer using a forward-viewing linear echoendoscope. Endoscopy 2016; 48 Suppl 1: E283-284.

74. Kamitani S, Fuchimoto S, Naomoto Y et al. A case of colonic metastasis of breast cancer positive for estrogen receptor. Hiroshima J Med Sci 1987; 36: 163-167.

75. Katz H, Jafri H, Saad R et al. Colonic Obstruction from an Unusual Cause: A Rare Case of Metastatic Invasive Ductal Carcinoma to the Colon. Cureus 2018; 10: e2588.

76. Khan AH, Thompson CC, Carr-Locke DL. Chronic diarrhea due to metastatic breast cancer. MedGenMed 2005; 7: 17.

77. Khan IM, R.; Khan, A.; Assad, S.; Zahid, M.; Sohail, M.S.; Yasin, F.; Qavi, A.H. Breast cancer metastases to the gastrointestinal tract presenting with anemia and intra-abdominal bleed. Cureus 2017; 9: e1429.

78. Kilgore TG, A.; Behctold, M.; Miick, R.; Diaz-Arias, A.; Ibdah, J.; Bragg, J. Breast cancer metastasis to the colon; a case report and review of the literature. Internet J Gastroenterol 2007; 6: 1-4.

79. Kim DHL, I.K.; Oh, C.H.; Lee, Y.S.; Park, J.K.; Park, W.C.; Jeon, H.M.; Byun, J.H.; Park, G. S.; Chang, S.K. [Colon obstruction due to colonic metastasis of a breast carcinoma]. J Korean Soc Coloproctol 2008; 24: 144-147.

80. Kim HW, Moon DH. Sigmoid colon metastasis from metaplastic breast carcinoma mimicking primary sigmoid colon cancer. Rev Esp Med Nucl Imagen Mol 2015; 34: 211-212.

81. Klein MS, Sherlock P. Gastric and colonic metastases from breast cancer. Am J Dig Dis 1972; 17: 881-886.

82. Koleilat I, Syal A, Hena M. Metastatic male ductal breast cancer mimicking obstructing primary colon cancer. Int J Biomed Sci 2010; 6: 66-70.

83. Koop H, Dombrowski H, Maroske D et al. [Segmental colonic stenosis in intestinal metastasis of breast carcinoma. A contribution to the differential diagnosis of colitis]. Dtsch Med Wochenschr 1988; 113: 1101-1104.

84. Koos L, Field RE. Metastatic carcinoma of breast simulating Crohn's disease. Int Surg 1980; 65: 359-362.

85. Koutsomanis D, Renier JF, Ollivier R et al. Colonic metastasis of breast carcinoma. Hepatogastroenterology 2000; 47: 681-682.

86. Krug B, Beyer D, Gunther D. [Metastasis of breast carcinoma to the intestinal tract and endometrium]. Rofo 1986; 145: 732-734.

87. Landi F, Marti Gallostra M, Espin Basany E et al. [Colon metastasis of lobular breast carcinoma]. Cir Esp 2012; 90: 470-471.

88. Laoutliev B, Harling H, Neergaard K, Simonsen L. Rectal metastasis from infiltrating lobular breast carcinoma: imaging with 18F-FDG PET. Eur Radiol 2005; 15: 186-188.

89. Lasson AK, S. Abdomino-perineal resection of the rectum for metastatic scirrhous breast carcinoma. Coloproctol 1982; 82: 186-188.

90. Lau LC, Wee B, Wang S, Thian YL. Metastatic breast cancer to the rectum: A case report with emphasis on MRI features. Medicine (Baltimore) 2017; 96: e6739.

91. Lauvin R, Iskandar MJ, Khayat D et al. [Intracolonic metastases of breast adenocarcinoma. A rare or unknown entity?]. Presse Med 1986; 15: 2116-2117.

92. Law WL, Chu KW. Scirrhous colonic metastasis from ductal carcinoma of the breast: report of a case. Dis Colon Rectum 2003; 46: 1424-1427.

93. Le Bouedec G, Kauffmann P, Darcha C et al. [Intestinal metastases from breast cancer. Apropos of 8 cases]. Ann Chir 1993; 47: 342-347.

94. Letessier E, Fignon A, Zachar D et al. [Linitis plastica of the colon secondary to breast cancer. Report of a case]. Ann Chir 1987; 41: 539-542.

95. Lin CC, Lin CC, Chen WS et al. Metastatic Malignancy to the Colon and Rectum: A Report of 14 Cases from One Single Institute. Dig Surg 2018; 35: 261-265.

96. Loh ZJL, K.T.; Chung, W.P.; Chen, W.C.; Kuo, H.L.; Chen, P.J.; Lu, H.H.; Hsu, H.P. Invasive lobular carcinoma of breast with synchronous colon metastasis. Formos J Surg 2017; 50: 69-73.

97. Lopez Deogracias M, Flores Jaime L, Arias-Camison I et al. Rectal metastasis from lobular breast carcinoma 15 years after primary diagnosis. Clin Transl Oncol 2010; 12: 150-153.

98. Lortholary A, Humeau B, Castanie H et al. [Lobular breast cancer metastasis to the colon]. Presse Med 2017; 46: 126-128.

99. Macias-Garcia F, Sobrino-Faya M, Dominguez-Munoz JE. Metastasis of lobular breast carcinoma diagnosed by rectal macrobiopsies. Rev Esp Enferm Dig 2010; 102: 660-661.

100. Maekawa H, Fujikawa T, Tanaka A. Successful laparoscopic investigation and resection of solitary colonic metastasis from breast cancer (with video). BMJ Case Rep 2012; 2012.

101. Malhotra A, Guturu P, Basim MS, Raju GS. A rare case of breast cancer metastasis presenting as linitis plastica of the stomach and colon (with videos). Gastrointest Endosc 2009; 70: 552-553; discussion 553.

102. Martin Perez E, Cardenoso P, Mancheno A et al. [Intestinal obstruction caused by solitary colonic metastasis of breast carcinoma]. Rev Esp Enferm Dig 1998; 90: 818-819.

103. Martin R, Mathews W, Scarcliff S. A rare presentation of breast cancer: near obstructing rectal mass and gastric outlet obstruction. J Surg Case Rep 2016; 2016.

104. Martinez Lesquereux L, Paredes Cotore JP, Ladra Gonzalez MJ, Beiras Torrado A. [Colon metastasis of lobular breast cancer]. Cir Esp 2010; 88: 122-124.

105. Martins Figueiredo LH, D.V.; Reis, J.A. A rare presentation of breast cancer. GE Port J Gastroenterol 2019; 26: 438-440.

106. Matsuda I, Matsubara N, Aoyama N et al. Metastatic lobular carcinoma of the breast masquerading as a primary rectal cancer. World J Surg Oncol 2012; 10: 231.

107. Matsumori MT, T. ; Tsuji, F.; et al. A case of breast cancer surviving long-term following various types of metastatic recurrence after radical surgery. . Geka 1987; 49: 396-399.

108. Matsuo SS, S,; Tsutsumi, R.; Azuma, T.; Yamaguchi, S. ; Hayashi, T. Recurrent breast cancer presenting as ureteral and colonic metastases. Acta Med Nagasaki 2007; 52: 35-37.

109. McLemore EC, Pockaj BA, Reynolds C et al. Breast cancer: presentation and intervention in women with gastrointestinal metastasis and carcinomatosis. Ann Surg Oncol 2005; 12: 886-894.

110. Melnick GS, Rosenholtz MJ. Metastatic breast carcinoma simulating ulcerative colitis. Report of a case. Am J Roentgenol Radium Ther Nucl Med 1961; 86: 702-706.

111. Michalopoulos A, Papadopoulos V, Zatagias A et al. Metastatic breast adenocarcinoma masquerading as colonic primary. Report of two cases. Tech Coloproctol 2004; 8 Suppl 1: s135-137.

112. Mistrangelo M, Cassoni P, Mistrangelo M et al. Obstructive colon metastases from lobular breast cancer: report of a case and review of the literature. Tumori 2011; 97: 800-804.

113. Molina-Barea R, Rios-Peregrina RM, Slim M et al. Lobular breast cancer metastasis to the colon, the appendix and the gallbladder. Breast Care (Basel) 2014; 9: 428-430.

114. Momiyama NA, I.; Nobuyuki, U.; et al. Five cases of laparotomy for gastrointestinal metastases of breast cancer. Nyugan no Nozomu 2003; 18: 272-276.

115. Motos-Mico J, Ferrer-Marquez M, Belda-Lozano R et al. Metastasis of lobular breast carcinoma in the sigmoid colon. Rev Esp Enferm Dig 2014; 106: 366-367.

116. Mourra N, Jouret-Mourin A, Lazure T et al. Metastatic tumors to the colon and rectum: a multi-institutional study. Arch Pathol Lab Med 2012; 136: 1397-1401.

117. Mroz A, Kiedrowski M. An unusual case of colonic adenocarcinoma development in the region of disseminating lobular breast carcinoma infiltration: diagnostic approach and review of the literature. Int J Clin Exp Pathol 2015; 8: 7470-7474.

118. Muehlenberg K, Dietl O, Piso P, Pech O. [Multiple smooth colon stenosis in 76-year-old female patient]. Internist (Berl) 2015; 56: 1191-1195.

119. Murukutla SV, A.; Paramanathan, K.; Kong, F.; Varma, S. Isolated colonic metastasis from primary invasive ductal brest carcinoma; role of tumor marker in early diagnosis. J Med Cases 2012; 3: 49-53.

120. Nair MS, Phillips BL, Navaratnam R, Fafemi O. Anorectal metastasis from breast carcinoma. J Gastrointest Cancer 2013; 44: 106-107.

121. Naomoto YF, S.; Kamitani, S.; Matsuda, T.; Gohchi, A.; Orita, K. [Colonic metastasis of breast cancer positive for estrogen receptor - a case report]. Nippon Daicho Komonbyo Gakki Zasshi 1987; 40: 172-176.

122. Nejati PS, M.; Amirian, F.; Ramezani, M. Metastatic breast cancer to colon: an unusual site of metastasis with a review of literature. Biomed. Res. Ther. 2019; 6.

123. Ng CE, Wright L, Pieri A et al. Rectal metastasis from Breast cancer: A rare entity. Int J Surg Case Rep 2015; 13: 103-105.

124. Nieboer P, van der Graaf WT, de Knegt RJ, van Dullemen HM. Rectal syndrome as first presentation of metastatic breast cancer. Am J Gastroenterol 2000; 95: 2138-2139.

125. Nikkar-Esfahani A, Kumar BG, Aitken D, Wilson RG. Metastatic breast carcinoma presenting as a sigmoid stricture: report of a case and review of the literature. Case Rep Gastroenterol 2013; 7: 106-111.

126. Nikolic I, Ivkovic-Kapicl T, Kukic B et al. Uncommon metastatic site from breast cancer. Vojnosanit Pregl 2012; 69: 806-808.

127. Norfaidhi Akram MNS, T.; Norly, S.; Rosani, P. Colonic metastasis from an invasive lobular carcinoma of the breast. J Surg Acad 2017; 7: 37-38.

128. Ohyama MY, H.; Fujino, Y.; Tominaga, M. [A case of rectal metastasis from breast cancer]. NRGGZ 2015; 76: 2125-2129.

129. Okamura S, Yanagisawa T, Ohishi K et al. [Right Hemi-Colectomy for a Metastatic Transverse Colon Tumor from Breast Cancer Following Bilateral Breast Cancer Resection - A Case Report]. Gan To Kagaku Ryoho 2016; 43: 2047-2049.

130. Okawa YM, K.; Taguchi, K.; Kikuchi, K.; Takeda, K.; Sano, H. [A case of colon metastasis from breast cancer diagnosed by laparoscopic biopsy]. Jap J Clin Surg 2006; 67: 2136-2141.

131. Okido M, Seo M, Hamada Y et al. Metastatic breast carcinoma simulating linitis plastica of the colon: report of a case. Surg Today 2011; 41: 542-545.

132. Okuma TA, S.; Matsumoto, T.; Hongoh, H.; Mita, S.; Kanemitsu, K. [A case of rectal metastasis due to breast cancer diagnosed about eight years after surgery]. Nihon Rinsho Geka Gakkai Zasshi 2008; 69: 2813-2817.

133. Okumura GK, H.; Yabuki, Y.; Tobbe, N.; Tanaka, K.; Hagiwara, M. [A case of rectal metastasis of breast cancer]. Nihon Rinsho Geka Gakkai Zasshi 2003; 64: 3109-3112.

134. Ortoleva A, ; Erra, S. Breast cancer metastatic in colonic mucosa: report of a case. Working Paper of Public Health 2013; 18: 1-7.

135. Osaku T, Ogata H, Magoshi S et al. Metastatic nonpalpable invasive lobular breast carcinoma presenting as rectal stenosis: a case report. J Med Case Rep 2015; 9: 88.

136. Paz A, Rath-Wolfson L, Wolloch Y. [Late solitary metastases of breast origin presenting as primary colonic carcinoma]. Harefuah 1996; 130: 9-10, 72.

137. Peinado BA, E. ; Pascual, I.; Rubio, I.; Alvarez, M.; Marijuan, J.L.; Diaz, J. . Gastrointestinal metastases from breast cancer: a case report. NHCCCR 2017; 1: 1-28.

138. Pendse AA, Edgerly CH, Fedoriw Y. Hemolytic anemia and metastatic carcinoma: case report and literature review. Lab Med 2014; 45: 132-135.

139. Pla VB, E.: Pallas, A.; Safont, M.J.; Roig, J.V. [Metastases from infiltrating ductal carcinoma of the breast mimicking primary obstructive colon cancer]. Cir Esp 2002; 71: 257-258.

140. Precetti FAL, C.; Marini, E.J.; Ghiraldo, A.L.; Gonzalez, P. Pietrantonio, A,; Lucatelli, N. [Colon metastases in primary breast ductal carcinoma]. Acta Gastroenterol Latinoam 2015; 45: 307-311.

141. Puapong DPG, R.; Lentz, S.E.; Alshak, N.S.; Abbas, M.A. Invasive ductal breast cancer metastatic to the rectum. Surg Rounds 2008; 1-4.

142. Pulanic R, Jelavic M, Premuzic M et al. [Breast cancer metastases to the stomach and colon: two case reports]. Lijec Vjesn 2012; 134: 159-163.

143. Rabau MY, Alon RJ, Werbin N, Yossipov Y. Colonic metastases from lobular carcinoma of the breast. Report of a case. Dis Colon Rectum 1988; 31: 401-402.

144. Rajan SSS, M.; Mestrah, M. Ductal carcinoma of the breast metastatisizing to the rectum. JSCR 2012; 5: 1-5.

145. Razzetta F, Tassara E, Saro F et al. Rare abdominal metastases from occult lobular breast cancer: report of two cases. Updates Surg 2011; 63: 129-133.

146. Rees BI, Okwonga W, Jenkins IL. Intestinal metastases from carcinoma of the breast. Clin Oncol 1976; 2: 113-119.

147. Rosati G, Ferrara D, Scarano E, Siciliano P. Colon and muscle metastases from lobular breast carcinoma: a very rare entity. Breast J 2012; 18: 77-79.

148. Ruiz S, Vilades C, Larios M, Luisa Diaz M. [Gastric and rectal linitis plastica presented as metastasic breast ductal carcinoma]. Med Clin (Barc) 2009; 132: 37-38.

149. Ruymbeke H, Harlet L, Stragier B et al. Anorectal metastasis from breast carcinoma: a case report and review of the literature. BMC Res Notes 2018; 11: 268.

150. Salazar-Campos JEL-H, M.E.; Nieto-Coronel, T.; Diaz-MOlina, R.; Cantu de Leon, D.; Vazquez-Romo, R. [Unusual colonic metastasis from primary breast cancer]. Gac Mex Oncol 2018; 17: 75-81.

151. Samo S, Sherid M, Husein H et al. Metastatic infiltrating ductal carcinoma of the breast to the colon: a case report and literature review. Case Rep Gastrointest Med 2013; 2013: 603683.

152. Samra B, Ghanem S, Ilyas G, Taiwo E. Screening Colonoscopy Unmasking Colonic Metastasis from an Occult Breast Ductal Carcinoma: A Case Report and Review of the Literature. Case Rep Oncol Med 2019; 2019: 8432079.

153. Sanmugachandran VA, D.; Wahab, M.Y.A. Colonic and small bowel metastasis from ductal carcinoma of the breast: a case report. Med J Malaysia 2013; 68.

154. Santini D, Altomare A, Vincenzi B et al. An increase of CA 19.9 as the first clinical sign of ileocecal valve metastasis from breast cancer. In Vivo 2006; 20: 165-168.

155. Saranovic D, Kovac JD, Knezevic S et al. Invasive lobular breast cancer presenting an unusual metastatic pattern in the form of peritoneal and rectal metastases: a case report. J Breast Cancer 2011; 14: 247-250.

156. Sasaki HO, Y.; Kawasai, K.; et al. [A case of invasive lobular carcinoma of the breast with rectal and gastric metastases]. Nichirin-gai kaishi 2013; 74: 362-366.

157. Savanis G, Simatos G, Tzaida O et al. Gastrointestinal tract metastasis as first presentation of breast cancer. J BUON 2006; 11: 79-81.

158. Sazuka T, Kimura M, Ikeda Y et al. [A Case of Colon Metastasis from Invasive Lobular Carcinoma of the Breast]. Gan To Kagaku Ryoho 2018; 45: 2473-2475.

159. Sazuka T, Miyazawa Y, Tochigi T et al. [A Case of Rectal Metastasis from Breast Cancer Diagnosed Two Years after Surgery]. Gan To Kagaku Ryoho 2017; 44: 1254-1256.

160. Schellenberg AE, Wood ML, Baniak N, Hayes P. Metastatic ductal carcinoma of the breast to colonic mucosa. BMJ Case Rep 2018; 2018.

161. Schwarz RE, Klimstra DS, Turnbull AD. Metastatic breast cancer masquerading as gastrointestinal primary. Am J Gastroenterol 1998; 93: 111-114.

162. Shakoor MT, Ayub S, Mohindra R et al. Unique presentations of invasive lobular breast cancer: a case series. Int J Biomed Sci 2014; 10: 287-293.

163. Shim JHS, E.J.; Lim, B.J.; Youk, J.H.; Kim, J.A.; Jeong, J. Localized metastasis to small and large bowel from breast cancer: a case report. J Korean Soc Radiol 2010; 62: 551-554.

164. Shimonov M, Rubin M. Metastatic breast tumors imitating primary colonic malignancies. Isr Med Assoc J 2000; 2: 863-864.

165. Signorelli C, Pomponi-Formiconi D, Nelli F, Pollera CF. Single colon metastasis from breast cancer: a clinical case report. Tumori 2005; 91: 424-427.

166. Smith JLP, R.W.; Bermann, M.M. Breast carcinoma: colonic metastases with perforation - report of two cases. Contemp Surg 1989; 35: 47-52.

167. Suda TK, H.; Ota, D.; Serizawa, H.; Kono, N.; Aoki, T. [A case of breast cancer with metastasis to the rectum 11 years after surgey which was controlled by hormone therapy for a long time. Nihon Rinsho Geka Gakkai Zasshi 2007; 68: 302-307.

168. Szabo J, Falkus B, Simon E et al. [Late gastrointestinal metastases of invasive lobular breast carcinoma mimicking Crohn's disease]. Orv Hetil 2010; 151: 1666-1671.

169. Taal BG, den Hartog Jager FC, Steinmetz R, Peterse H. The spectrum of gastrointestinal metastases of breast carcinoma: II. The colon and rectum. Gastrointest Endosc 1992; 38: 136-141.

170. Tahir MS, A.; Singh, A. Can metastatic breast cancer spread to the colon? J Cna Sci Res 2017; 3.

171. Takeuchi H, Hiroshige S, Yoshikawa Y et al. A case of synchronous metastasis of breast cancer to stomach and colon. Anticancer Res 2012; 32: 4051-4055.

172. Tamura HS, K.; Maeda, S.; Ikeda, N.; Kojima, M.; Saito, K. [Two cases of advanced and recurrent breast cancer (invasive lobular carcinoma) with metastatic rectal stenosis]. Nihon Rinsho Geka Gakkai Zasshi 2005; 66: 41-45.

173. Theraux J, Bretagnol F, Guedj N et al. Colorectal breast carcinoma metastasis diagnosed as an obstructive colonic primary tumor. A case report and review of the literature. Gastroenterol Clin Biol 2009; 33: 1114-1117.

174. Titi MA, Anabtawi A, Newland AD. Isolated gastrointestinal metastasis of breast carcinoma: a case report. Case Rep Med 2010; 2010: 615923.

175. Tohfe M, Shami P, Aftimos G, Saade M. Gastrointestinal metastases from breast cancer: a case report. South Med J 2003; 96: 624-625.

176. t'Sjoen P, Andre R, Van den Heulen B, Musin L. [Colonic metastases of breast cancer. Apropos of a case]. J Belge Radiol 1984; 67: 411-415.

177. Tsujimura K, Teruya T, Kiyuna M et al. Colonic metastasis from breast carcinoma: a case report. World J Surg Oncol 2017; 15: 124.

178. Uskent NB, H.; Cakmakci, M.; Saglam, S.; Koksal, U. Breast cancer metastases tot the stomach and colon mimicking primary gastrointestinal cancer: four cases and literature review. Adv Mod Oncol Res 2016; 2: 204-210.

179. Uygun K, Kocak Z, Altaner S et al. Colonic metastasis from carcinoma of the breast that mimics a primary intestinal cancer. Yonsei Med J 2006; 47: 578-582.

180. Vaidya JS, Mukhtar H, Bryan R. Colonic metastasis from a breast cancer--a case report and a few questions. Eur J Surg Oncol 2002; 28: 463-464.

181. van Halteren HK, Peters H, Gerlag PG. Large bowel mucosal metastases from breast cancer. J Clin Oncol 1998; 16: 3711-3713.

182. Venturini F, Gambi V, Di Lernia S et al. Linitis Plastica of the Rectum As a Clinical Presentation of Metastatic Lobular Carcinoma of the Breast. J Clin Oncol 2016; 34: e54-56.

183. Villa Guzman JC, Espinosa J, Cervera R et al. Gastric and colon metastasis from breast cancer: case report, review of the literature, and possible underlying mechanisms. Breast Cancer (Dove Med Press) 2017; 9: 1-7.

184. Voravud N, el-Naggar AK, Balch CM, Theriault RL. Metastatic lobular breast carcinoma simulating primary colon cancer. Am J Clin Oncol 1992; 15: 365-369.

185. Wagner J, Nerlich A, Schepp W. [Diffuse mucosal gastric and coecal metastasis from occult breast cancer]. Dtsch Med Wochenschr 2009; 134: 879-882.

186. Wang G, Wang T, Jiang J et al. Gastrointestinal tract metastasis from tubulolobular carcinoma of the breast: a case report and review of the literature. Onco Targets Ther 2014; 7: 435-440.

187. Washington K, McDonagh D. Secondary tumors of the gastrointestinal tract: surgical pathologic findings and comparison with autopsy survey. Mod Pathol 1995; 8: 427-433.

188. Watanabe M, Ishibashi O, Maeda M et al. [A Case of Gastrointestinal Metastases of Breast Cancer Effectively Treated with Gemcitabine and Paclitaxel Combination Chemotherapy]. Gan To Kagaku Ryoho 2015; 42: 985-987.

189. Weisberg A. Metastatic adenocarcinoma of the breast masquerading as Crohn's disease of the colon. Am J Proctol Gastroenterol Colon Rectal Surg 1982; 33: 10-15, 22.

190. Wiisanen JM, Kaur JS. Gastrointestinal Metastases from Breast Cancer, a Comprehensive Review. Breast J 2015; 21: 572-573.

191. Wiisanen JMK, J.S. Gastrointestinal metastase from breast cancer, a diagnostic dilemma. MOJ Clin Med Case Rep 2015; 2: 27-30.

192. Winston CB, Hadar O, Teitcher JB et al. Metastatic lobular carcinoma of the breast: patterns of spread in the chest, abdomen, and pelvis on CT. AJR Am J Roentgenol 2000; 175: 795-800.

193. Xue F, Liu ZL, Zhang Q et al. Mesorectum localization as a special kind of rectal metastasis from breast cancer. World J Gastroenterol 2015; 21: 4408-4412.

194. Yanagisawa K, Yamamoto M, Ueno E, Ohkouchi N. Synchronous rectal metastasis from invasive lobular carcinoma of the breast. J Gastroenterol Hepatol 2007; 22: 601-602.

195. Yokota T, Kunii Y, Kagami M et al. Metastatic breast carcinoma masquerading as primary colon cancer. Am J Gastroenterol 2000; 95: 3014-3016.

196. You Q, Fang Y, Li C et al. Multiple metastases of bones and sigmoid colon after mastectomy for ductal carcinoma in situ of the breast: a case report. BMC Cancer 2019; 19: 844.

197. Zhang B, Copur-Dahi N, Kalmaz D, Boland BS. Gastrointestinal manifestations of breast cancer metastasis. Dig Dis Sci 2014; 59: 2344-2346.

198. Zhou XC, Zhou H, Ye YH et al. Invasive ductal breast cancer metastatic to the sigmoid colon. World J Surg Oncol 2012; 10: 256.

199. Endo MA, T.; Tsuchida, A.; Aoki, T. [A case of breast cancer with rectal metastasis]. Nihon Rinsho Geka Gakkai Zasshi 2010; 71: 52-56.

200. Jones AK, M.R.; Justice, A.; Navarro, F. Colonic metastasis from infiltrating ductal breast carcinoma in a male patient: a case report. Int J Surg Case Rep 2019; 54: 34-38.

201. Bando MT, K.; Sumi, Y.; Azuma, M.; Yoshida, N.; Oda, Y.; Kasai, K.; Ozeki, Y. [A case of breast cancer causing intestinal metastases]. Nihon Rinsho Geka Gakkai Zasshi 1999; 60: 50-55.

**Supplemental figure**


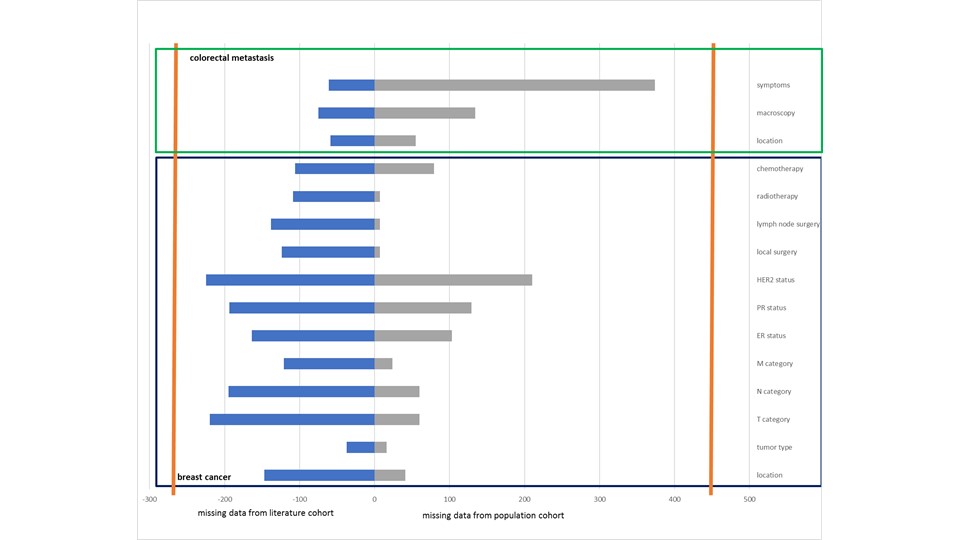

Supplement: Supplementary file 1 — Supplementary file1 (DOCX 187 kb)—Completeness of the variables per cohort. [file 10585_2023_10207_MOESM1_ESM.docx]
